# *QuickStats*: Age-Adjusted* Trends in the Prevalence of Herpes Simplex Virus Type 1 (HSV-1) and Herpes Simplex Virus Type 2 (HSV-2) Among Adolescents and Adults Aged 14–49 Years — United States, 1999–2000 Through 2015–2016

**DOI:** 10.15585/mmwr.mm6706a7

**Published:** 2018-02-16

**Figure:**
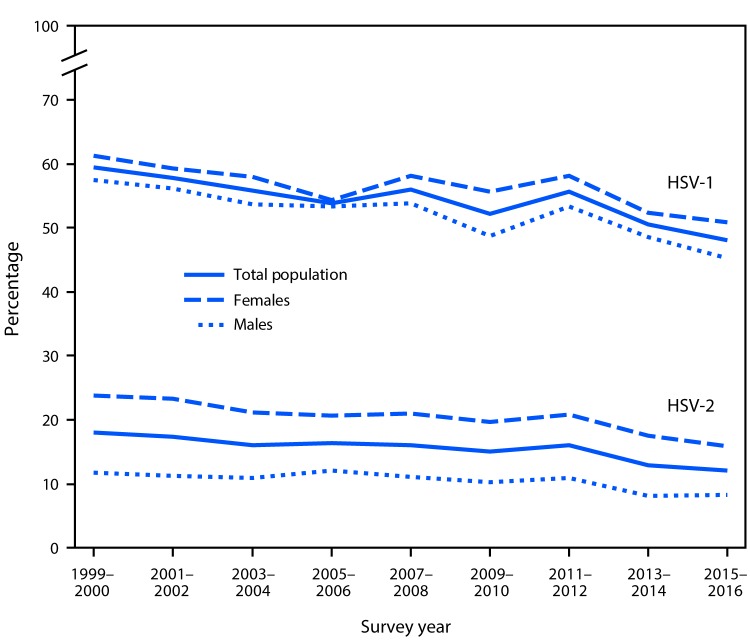
During 2015–2016, the age-adjusted prevalence of HSV-1 was 48.1% among adolescents and adults aged 14–49 years (50.9% for females and 45.2% for males). Prevalence was higher for females than males in most 2-year periods from 1999–2000 to 2015–2016. Also during 2015–2016, the age-adjusted prevalence of HSV-2 for those aged 14–49 years was 12.1% (15.9% among females compared to 8.2% among males) and was higher for females than males for all 2-year periods. Prevalence significantly declined from 1999–2000 through 2015–2016 for HSV-1 and HSV-2 among both males and females.

**Source:** NCHS Data Brief No. 304. https://www.cdc.gov/nchs/products/databriefs/db304.htm.

